# Utility of TTR-INR guided warfarin adjustment protocol to improve time in therapeutic range in patients with atrial fibrillation receiving warfarin

**DOI:** 10.1038/s41598-024-61664-5

**Published:** 2024-05-22

**Authors:** Paisit Kosum, Noppachai Siranart, Kunathip Nissaipan, Wattakorn Laohapiboolrattana, Walit Sowalertrat, Kanittha Triamamornwooth, Rawiwan Arunphan, Apiradee Sriyom, Voravut Rungpradubvong

**Affiliations:** 1 Division of Cardiovascular Medicine, Department of Medicine, Faculty of Medicine, Chulalongkorn University, Bangkok, Thailand; 2 Division of Cardiovascular Medicine, Department of Medicine, Faculty of Medicine, Naresuan University, Phitsanulok, Thailand; 3 Faculty of Medicine, Chulalongkorn University, Bangkok, Thailand; 4 Clinical Pharmacokinetics and Pharmacogenomics Research Unit, Faculty of Medicine, Chulalongkorn University, Bangkok, Thailand; 5 Department of Pharmacology, Faculty of Medicine, Chulalongkorn University, Bangkok, Thailand; 6 Division of Neurology, Department of Medicine, Faculty of Medicine, Naresuan University, Phitsanulok, Thailand; 7 Warfarin Clinic, King Chulalongkorn Memorial Hospital, Thai Red Cross Society, Bangkok, Thailand; 8 Cardiac Center, King Chulalongkorn Memorial Hospital, Thai Red Cross Society, Bangkok, 10330 Thailand

**Keywords:** Atrial fibrillation (AF), Time in therapeutic range (TTR), International normalized ratio (INR), Vitamin K antagonist (VKA), Oral anticoagulants (OACs), Non-vitamin K antagonist oral anticoagulants (NOACs), Cardiology, Atrial fibrillation

## Abstract

Warfarin remains the most prescribed oral anticoagulant of choice in atrial fibrillation (AF) patient in resource-limited settings. Despite evidence linking Time in Therapeutic Range (TTR) to patient outcomes, its use in clinical practice is not widespread. This prospective study explores the impact of a TTR-INR guided Warfarin adjustment protocol on TTR in AF patients. Conducted at the Warfarin clinic of King Chulalongkorn Memorial Hospital. TTR was calculated using the Rosendaal linear interpolation method at baseline, and then at 6 and 12 months post-protocol implementation. The primary outcome was the improvement in TTR following the protocol’s implementation. The study analyzed 57 patients, with a mean age of 72 years and an even gender distribution. At baseline, 53% of patients had a TTR of less than 65%. However, TTR significantly improved from 65% at baseline to 80% after 12 months of protocol implementation (*p* < 0.001). Furthermore, there was a significant increase in the proportion of patients with a TTR of 65% or more, from 47 to 88% (*p* < 0.001). During the follow-up period in the first 12 months, three patients died, but no ischemic or major bleeding events occurred. The significant improvement in TTR after 12 months of protocol implementation suggests that this strategy could provide additional value in improving TTR and outcomes in AF patients receiving Warfarin.

## Introduction

Atrial Fibrillation (AF) is a common cardiac arrhythmia, affecting approximately 1–2% of the general population. It is associated with an increased risk of thrombotic events in the neurological system, especially stroke^1–4^. Anti-coagulant therapy is a standard of practice for ischemic stroke prevention in patients with AF. n Thailand, warfarin, a widely available vitamin K antagonist (VKA), is the standard anticoagulant therapy for ischemic stroke prevention in AF patients. However, studies have shown that non-vitamin K antagonist oral anticoagulants (NOACs) are at least non-inferior, if not superior, to warfarin in stroke prevention^5^, with a better safety profile^6^. Interestingly, NOACs are not included in the Thailand National List of Essential Medicines (NLEM), while warfarin is listed. This discrepancy poses challenges in clinical practice for ischemic event prevention in AF patients in Thailand.

Time in Therapeutic Range (TTR), calculated from International Normalized Ratio (INR) measurements, reflects how well warfarin is managed. Patients with TTR less than 60% have higher mortality rates and bleeding complications, while those with higher TTR have better survival rates^7,8^. The accepted target TTR is 65–70% or higher for optimal clinical outcomes with fewer complications^6,9^.

Despite studies advocating for the use of TTR in monitoring patients on Vitamin K Antagonists (VKA), with a target range of 65–70% or higher, its adoption has been limited due to a lack of evidence supporting its benefits for Thai patients. Furthermore, the inability to switch from VKAs to Novel Oral Anticoagulants (NOACs) due to restrictions in the NLEM and socioeconomic factors of patients, warfarin remains the primary anticoagulant used in stroke prevention for patients with AF in Thailand.

The current standard for adjusting warfarin dosage is primarily based on the patient’s INR levels, without considering TTR. This research, however, introduces a novel approach: a TTR-INR guided warfarin adjustment protocol. This protocol, adapted from the Hamilton Dosing Nomogram and treatment guidelines endorsed by the Thai Heart Association for patients on oral anticoagulants, categorizes patients into two groups based on their initial TTR values:

- Patients with TTR levels below the standard threshold (TTR < 65%).
- Patients with TTR levels within or above the standard threshold (TTR ≥ 65%).

Upon implementing this protocol, its efficacy will be assessed by monitoring the changes in the number of patients achieving the standard TTR threshold (TTR ≥ 65%). The objective of this research is to evaluate the efficacy of warfarin dose adjustment guidelines, both before and after the incorporation of TTR and INR assistance. It is hypothesized that patients with TTR levels below the standard threshold (TTR < 65%) will experience an increase in TTR levels following warfarin dosage adjustments guided by the TTR-INR assisted protocol. This intervention is expected to lead to a decrease in thromboembolic events within the cerebral and peripheral vascular systems, and a subsequent reduction in hemorrhagic occurrences.

## Material and method

### Study design

This prospective cohort, non-randomized, single-center study was conducted in Warfarin Clinic, King Chulalongkorn Memorial Hospital, Bangkok, Thailand.

The study was approved by the Institutional Review Board of the Research Ethics Review Committee for Research Involving Human Research Participants, Health Sciences Group, Chulalongkorn University (COA No. 202/2021, IRB No. 899/63), and is in accordance with the Helsinki Declaration of 1983, was registered in Thai Clinical Trials Registry (TCTR20201224003, Date of registration: 24/12/2020).

### Patient enrollment

The study enrolled consecutive patients diagnosed with Non-Valvular Atrial Fibrillation (NVAF), confirmed by either a 12-lead ECG or an ECG strip showing at least 30 s of AF, including those recorded by wearable devices. These patients were receiving warfarin treatment and were part of a study conducted at King Chulalongkorn Memorial Hospital from February 1, 2021, to February 1, 2022.

The inclusion criteria required patients to be aged 18 years or older, have a minimum of 2 years of warfarin use, and have undergone at least 3 separate INR measurements. The exclusion criteria included significant valvular heart disease, pregnancy, atrial fibrillation associated with transient reversible causes, recent use of Novel Oral Anticoagulants (NOACs) within the past 2 years, and inability or refusal to attend follow-up appointments. All participants provided written informed consent prior to their participation in the study.

### TTR calculation and warfarin adjustment protocol

The percentage of time a patient maintained an International Normalized Ratio (INR) between 2 and 3 was determined using the Rosendaal linear interpolation method^7^. Patients were categorized into two groups based on their anticoagulant control:

- Poor anticoagulant control (Group 1): Defined as a Time in Therapeutic Range (TTR) less than 65%.
- Good anticoagulant control (Group 2): Defined as a TTR of 65% or higher.

All participants underwent warfarin dose adjustments according to the TTR-INR guided warfarin adjustment protocol, detailed in Table S5 of the supplementary appendix. The TTR was calculated at three different time points: at baseline, 6 months, and 12 months after the implementation of the protocol.

### Clinical follow-up and protocol assessment

Clinical follow-up was conducted for up to one year at the warfarin clinic, either during outpatient visits or through telephone interviews. The feasibility of the TTR-INR guided warfarin adjustment protocol was assessed using a questionnaire, and any protocol violations were recorded. The protocol’s accuracy, efficacy, and safety were evaluated by a team of two cardiologists and one hematologist. To ensure the reliability of the questionnaire used in the study, a reliability analysis was performed using Cronbach’s alpha coefficient. The analysis yielded a value of 0.85.

### Data collection

Baseline clinical characteristics, including demographic information, medical history, medication, left ventricular ejection fraction (LVEF), and laboratory tests (complete blood count, renal function tests, electrolytes, liver function tests, and INR) were collected from patient medical records. Ischemic and bleeding risk scores were calculated using the SAMe-TT2R2, CHA2DS2-VASc, and HAS-BLED scoring systems. Major bleeding, clinically relevant non-major bleeding (CRNM), and minor bleeding were categorized according to the 2005 International Society on Thrombosis and Haemostasis (ISTH) criteria. Renal disease, pulmonary disease, and liver disease were defined based on laboratory data and medical record documentation.

### Sample size calculation

To calculate the sample size for a census study aiming to detect a minimum clinically important difference of 20% in categorical data with two independent groups, the following parameters are used:

$${\text{N}} = \frac{{\left( {{\text{Z}}_{1 - \alpha /2} + {\text{Z}}_{1 - \beta } } \right)^{2} \left( {{\text{p}}_{1} (1 - {\text{p}}_{1} ) + {\text{p}}_{2} (1 - {\text{p}}_{2} )} \right)}}{{{\text{MCD}}^{2} }}$$

N = Sample size (93).

Z1-α/2 = 1.96 (for α = 0.05, two-tailed test).

Z1-β = 0.84 (for β = 0.2, power = 80%).

P1 = Proportion of patients with TTR ≥ 65% before protocol implementation (55%).

P2 = Proportion of patients with TTR ≥ 65% after protocol implementation (75%).

MCD = Minimal clinically important difference (20%), Drop-out rate = 10%

Given the study duration of up to 1 year and considering a potential drop-out rate of 10%, the sample size is adjusted to 93 individuals.

This sample size calculation ensures that the study will have sufficient power to detect the specified clinically important difference with a significance level of 0.05 and a power of 80%.

### Outcomes

The primary endpoint of this study was the change in Time in Therapeutic Range (TTR) after the implementation of the protocol.

The secondary endpoints included: the feasibility of the protocol and any violations that occurred, the proportion of patients who switched to using NOACs, the incidence of ischemic stroke, the occurrence of bleeding events, and all-cause mortality.

### Statistical analysis

Categorical data was presented as frequency and percentage. Continuous data was presented as mean ± standard deviation (SD) for normal distribution, and as median for skewed distribution.

Categorical data were compared using McNemar’s Chi-square test and continuous paired data was compared using paired t-test.

*P*-value < 0.05 was accepted as statistically significant. All statistical analysis was performed using SPSS version 28 statistical software.

### Ethical approval

Ethics approval was obtained from the Institutional Review Board and the Ethics Committee of Faculty of Medicine, Chulalongkorn University (COA No. 202/2021, IRB No. 899/63).

### Informed consent statement

Informed consent was obtained from all individual participants included in the study.

## Results

From February 1, 2021, to February 1, 2022, we screened a total of 95 consecutive patients with non-valvular atrial fibrillation (NVAF) at King Chulalongkorn Memorial Hospital, Bangkok. Out of these, 73 patients were undergoing treatment with warfarin and were thus enrolled in our study. However, 16 patients had to be excluded due to incomplete data. The reasons for exclusion were as follows: 10 were lost to follow-up, 2 declined to participate, 3 had reasons to target an INR of more than 2.0–3.0, and 1 had a high INR (> 4.99) at the enrollment date. A total of 57 NVAF patients who were treated with warfarin had complete data for TTR calculation were included in this study.
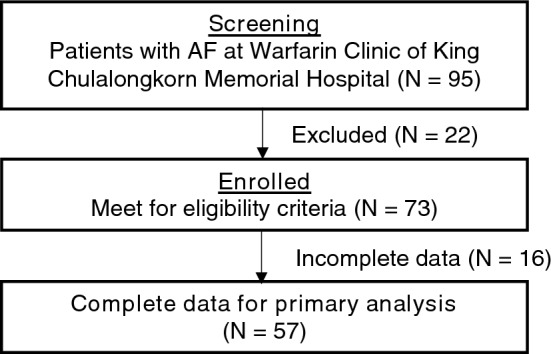


### Baseline characteristics

This study included 57 participants with an average age of 72 ± 11 years, 50% were female. The mean left ventricular ejection fraction (LVEF) was 56%, with 17% of the patients having an LVEF less than 40%. 32% of the patients reported ischemic heart diseases (IHD), 57% had hypertension, 36% had diabetes mellitus, 46% had dyslipidemia, and 21% had a history of stroke. Additionally, 21% had chronic kidney disease (CKD) at least at stage III, 1.8% had end-stage renal disease (ESRD), and 45% had a history of heart failure (HF) (Table 1).

**Table 1:** Baseline characteristics of the study population compared between patients with TTR ≥ 65% and patients with TTR < 65%

Characteristics	All patients (n = 57) N (%) or Mean ± SD	TTR < 65% (n = 30) N (%) or Mean ± SD	TTR ≥ 65% (n = 27) N (%) or Mean ± SD	*P*-value^1^
General information				
Age (years)	72 ± 11	73 ± 12	70 ± 10	0.278
Female	28 (50)	16 (48.48)	12 (52.17)	0.786
LV systolic function (LVEF)	55.98 ± 15.39	55.76 ± 16.18	56.37 ± 14.32	0.892
LVEF ≤ 40%	9 (17.31)	6 (18.18)	3 (15.79)	0.911
LVEF 41–49%	7 (13.46)	5 (15.15)	2 (10.53)	
LVEF ≥ 50%	36 (69.23)	22 (66.67)	14 (73.68)	
Medical history				
Heart failure	25 (44.64)	16 (48.48)	9 (39.13)	0.488
Diabetes mellitus	20 (35.71)	11 (33.33)	9 (39.13)	0.656
Coronary artery disease	18 (32.14)	11 (33.33)	7 (30.43)	0.819
Prior PCI	8 (14.29)	5 (15.15)	3 (13.04)	1.000
Prior CABG	5 (8.93)	2 (6.06)	3 (13.04)	0.392
Hypertension	32 (57.14)	17 (51.52)	15 (65.22)	0.308
Dyslipidemia	26 (46.43)	15 (45.45)	12 (52.17)	0.621
Old CVA	12 (21.43)	8 (24.24)	4 (17.39)	0.743
CKD stage ≥ 3	12 (21.43)	10 (30.30)	2 (8.70)	0.053
ESRD	1 (1.79)	1 (3.03)	0 (0)	1.000
PAD	1 (1.79)	1 (3.03)	0 (0)	1.000
Cirrhosis	2 (3.57)	1 (3.03)	1 (4.35)	1.000
COPD	2 (3.57)	1 (3.03)	1 (4.35)	1.000
OSA	4 (7.14)	1 (3.03)	3 (39.13)	0.295
Cancer	6 (10.71)	15 (15.15)	1 (4.35)	0.383
Cardiac implantable electronic device (CIED)				
PPM	9 (16.07)	6 (18.18)	3 (13.04)	0.607
ICD	2 (3.57)	2 (6.06)	0 (0)	0.507
CRT	0 (0)	0 (0)	0 (0)	–
Procedure				
AF RFA	3 (5.36)	1 (3.03)	2 (8.70)	0.562
AV node ablation	0 (0)	0 (0)	0 (0)	–
Medications				
Furosemide	28 (50)	18 (54.55)	10 (43.48)	0.415
Spironolactone	7 (12.50)	5 (15.15)	2 (8.70)	0.688
Statin	46 (82.14)	29 (87.88)	17 (73.91)	0.179
Beta blocker	41 (73.21)	25 (75.76)	16 (69.57)	0.607
ACEI	11 (19.64)	7 (21.21)	3 (39.13)	0.723
ARB	16 (28.57)	7 (21.21)	4 (17.39)	0.144
Sacubitril/Valsartan (ARNI)	2 (3.57)	2 (6.06)	0 (0)	0.507
Digoxin	7 (12.50)	5 (15.15)	2 (8.70)	0.688
Hydralazine	2 (3.57)	2 (6.06)	0 (0)	0.507
Nitrate	4 (7.14)	4 (12.12)	0 (0)	0.136
Warfarin (dose: mg/week)	21.02 ± 9.69 (Mean 18)	22.33 ± 11.12 (Mean 19.5)	19.13 ± 9.98 (Mean 18)	0.363
Aspirin	1 (1.79)	0 (0)	1 (4.35)	0.411
P2Y12 inhibitor (Clopidogrel)	1 (1.79)	1 (3.03)	0 (0)	1.000
Laboratory investigation				
Hemoglobin (g/dL)	12.66 ± 2.00	12.24 ± 2.10	13.27 ± 1.69	0.055
Hematocrit (%)	38.59 ± 5.69	37.36 ± 5.88	40.36 ± 5.00	0.055
Platelet (cu.mm^2^)	221,200 ± 70,560	229,180 ± 78,250	209,740 ± 57,530	0.315
Blood urea nitrogen (mg/dL)	20.31 ± 11.61	23.52 ± 13.75	15.50 ± 4.25	**0.010**
Creatinine (mg/dL)	1.23 ± 0.67	1.34 ± 0.82	1.06 ± 0.32	0.212
Sodium (mmol/L)	140.04 ± 2.40	139.70 ± 2.63	140.55 ± 1.95	0.314
Potassium (mmol/L)	4.11 ± 0.40	4.16 ± 0.41	4.03 ± 0.38	0.241
Chloride (mmol/L)	104.62 ± 3.27	104.15 ± 3.92	105.32 ± 1.81	0.144
Bicarbonate (mmol/L)	27.20 ± 2.93	27.24 ± 2.84	27.14 ± 3.12	0.897
Albumin (g/dL)	3.78 ± 0.72	3.73 ± 0.42	3.85 ± 1.01	**0.046**
Total bilirubin (mg/dL)	0.91 ± 0.54	0.97 ± 0.58	0.81 ± 0.45	0.353
Direct bilirubin (mg/dL)	0.44 ± 0.30	0.48 ± 0.34	0.37 ± 0.21	0.271
AST (SGOT) (U/L)	25.48 ± 9.75	25.36 ± 9.12	25.67 ± 10.90	0.901
ALT (SGPT) (U/L)	22.28 ± 11.17	20.15 ± 9.05	25.62 ± 13.44	0.126
ALP (U/L)	86.23 ± 39.27	94.36 ± 44.94	74.26 ± 25.64	0.068

^1^*P*-value for comparison between patients with TTR ≥ 65% and TTR < 65%

Significant values are in bold.

All patients were treated with warfarin, with an average dose of 21.02 ± 9.69 mg per week and a median dose of 18 mg per week. The average CHA2DS2-VASc score, HAS-BLED score, and SAMe-TT2R2 score were 3.82 ± 1.82, 2.13 ± 1.01, and 3.30 ± 0.74, respectively. The average TTR was 64.69 ± 20.14% (mean = 62.78%). Notably, 30 patients (52.63%) fell into the poor anticoagulant control group, with a TTR less than 65%. These findings are detailed in Tables 2 and 3.

**Table 2:** Risk scores of the study population compared between patients with TTR ≥ 65% and patients with TTR < 65%

Risk score	All patients (n = 57) N (%) or Mean ± SD	TTR < 65% (n = 30) N (%) or Mean ± SD	TTR ≥ 65% (n = 27) N (%) or Mean ± SD	*P* value^1^
CHA2DS2-VASc score				
Total score	3.82 ± 1.82	3.97 ± 1.90	3.61 ± 1.73	0.436
Number of positive each component				
Congestive heart failure/ LV dysfunction	25 (44.64)	19 (57.58)	6 (26.09)	0.020
Hypertension	36 (64.29)	18 (54.55)	18 (78.26)	0.068
Age > 75 years	24 (35.71)	16 (48.48)	8 (34.78)	0.308
Diabetes mellitus	20 (35.71)	12 (36.36)	8 (34.78)	0.903
Stroke/TIA/thromboembolism	11 (19.64)	6 (18.18)	5 (21.74)	0.742
Vascular disease	16 (28.57)	11 (33.33)	5 (21.74)	0.345
Age 65–74 years	19 (33.93)	11 (33.33)	8 (34.78)	0.910
Sex categrophy (female sex)	28 (50)	16 (48.48)	12 (52.17)	0.786
HAS-BLED score				
Total score	2.13 ± 1.01	2.39 ± 1.09	1.74 ± 0.75	0.016
Number of positive each component				
Hypertension	36 (64.29)	18 (54.55)	18 (78.26)	0.068
Abnormal liver or renal function	36 (64.29)	6 (18.18)	1 (4.35)	0.220
Stroke	11 (19.64)	6 (18.18)	5 (21.74)	0.742
Bleeding	2 (3.57)	2 (6.06)	0 (0)	0.507
Labile INR	22 (39.29)	22 (66.67)	0 (0)	< 0.001
Elderly (> 65 years)	39 (69.64)	24 (72.73)	15 (65.22)	0.548
Drug or alcohol	2 (3.57)	1 (3.03)	1 (4.35)	1.000
SAMe-TT2R2 score				
Total score	3.30 ± 0.74	3.21 ± 0.60	3.43 ± 0.90	0.249
Number of positive each component				
Sex (female)	28 (50)	16 (48.48)	12 (52.17)	0.786
Age (< 60 years)	7 (12.5)	4 (12.12)	3 (13.04)	1.000
Medical history	33 (58.93)	20 (60.61)	13 (56.52)	0.261
Treatment (rhythm control strategy)	1 (1.79)	0 (0)	1 (4.35)	0.411
Tobacco use (within 2 years)	0 (0)	0 (0)	0 (0)	–
Race (non-Caucasian)	56 (100)	33 (100)	23 (100)	–

^1^*P*-value for comparison between patients with TTR ≥ 65% and TTR < 65%

The CHA2DS2-VASc score calculates stroke risk for patients with AF, ranging from 0 to 9 (higher scores indicating a greater risk of stroke).

The HAS-BLED score predicts major bleeding risk and estimates the risk of major bleeding for patients on anticoagulation to assess the quality of atrial fibrillation care, also ranging from 0 to 9 (higher scores indicating a higher risk of major bleeding for patients on anticoagulation).

The SAMe-TT2R2 score aids decision-making between a non-VKA oral anticoagulant (NOAC) and a vitamin K antagonist (VKA). A score of 0–2 suggests the patient would benefit from a VKA, while a score > 2 indicates the use of alternative strategies (e.g., NOAC).

**Table 3:** Baseline TTR of the study population compared between patients with TTR ≥ 65% and patients with TTR < 65%

Baseline TTR	All patients (n = 57)	TTR < 65% (n = 30)	TTR ≥ 65% (n = 27)	*P*-value^1^
TTR (Mean ± SD)	64.69 ± 20.14	49.50 ± 13.15	81.56 ± 10.89	** < 0.001**
TTR (Median)	62.78	54.88	79.40	** < 0.001**

^1^P-value for comparison between patients with TTR ≥ 65% and TTR < 65.

Significant values are in bold.

In our study, patients in the poor anticoagulant control group (TTR less than 65%) had higher BUN and HAS-BLED scores compared to those in the good anticoagulant control group. Specifically, the BUN was 23.52 ± 13.75 versus 15.50 ± 4.25 (*p* = 0.01), and the HAS-BLED scores were 2.39 ± 1.09 versus 1.74 ± 0.75 (*p* = 0.016).

Furthermore, patients in the poor anticoagulant control group had a lower serum albumin level than those in the good control group (3.73 ± 0.42 versus 3.85 ± 1.01; *p* = 0.046). However, no significant differences were observed between the two groups in terms of age, sex, LVEF, number of prior cardiovascular diseases, drugs used (including statin, diuretic, beta-blocker, ACEI/ARB/ARNI, digoxin, hydralazine, nitrate, anticoagulant, aspirin, and P2Y12 inhibitor), CHA2DS2-VASc score, SAMe-TT2R2 score, history of Cardiac Implantable Electronic Devices (CIED), and other laboratory testing.

The baseline characteristics of the study population, compared between patients with TTR ≥ 65% and patients with TTR < 65%, are shown in Table 1.

### Primary outcome

**Figure 1:**
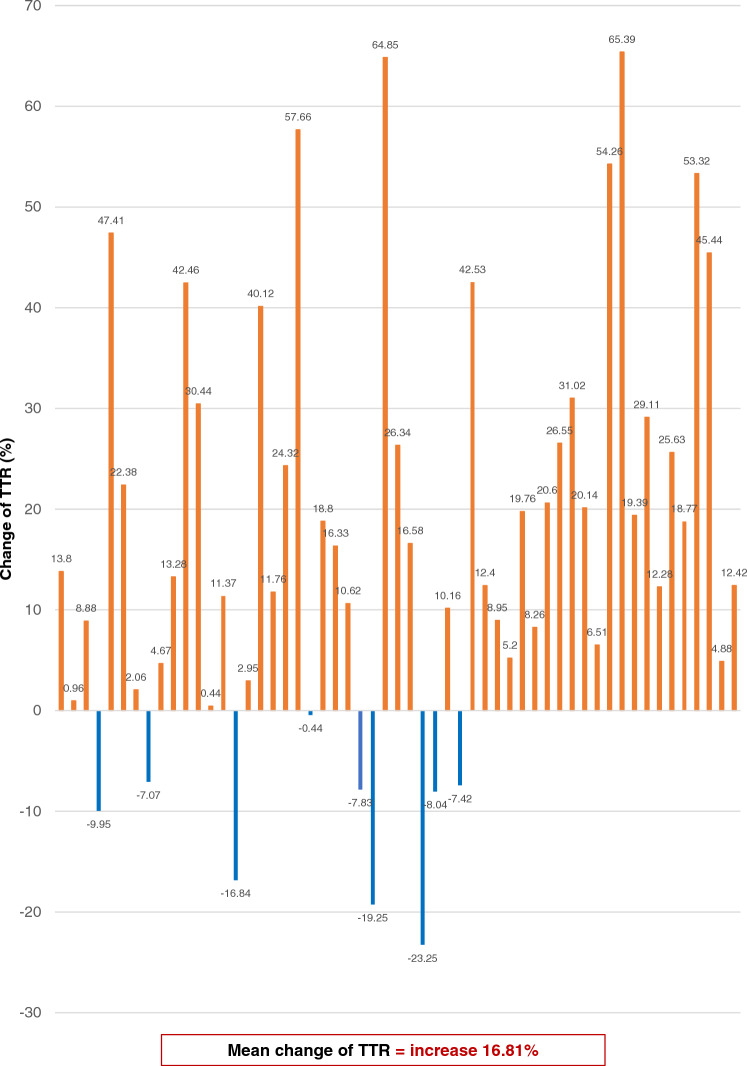
Individual change of TTR before and after 12 months of protocol implementation in 57 patients with non-valvular atrial fibrillation received warfarin.

**Figure 2:**
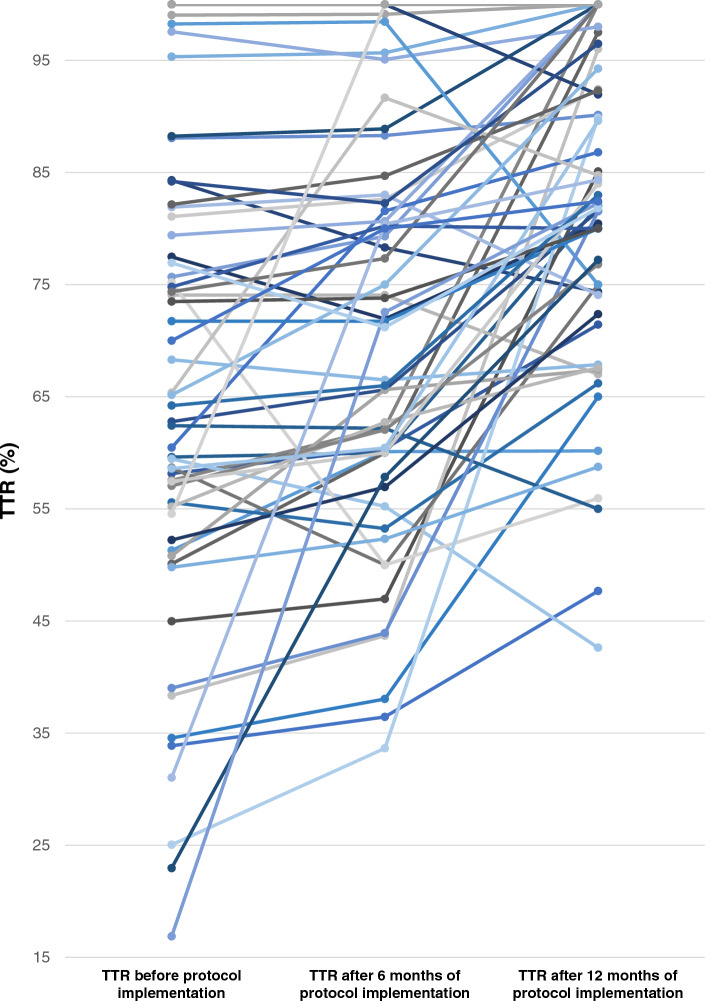
Individual change of TTR before and after 6 and 12 months of protocol implementation in 57 patients with non-valvular atrial fibrillation received warfarin.

**Figure 3:**
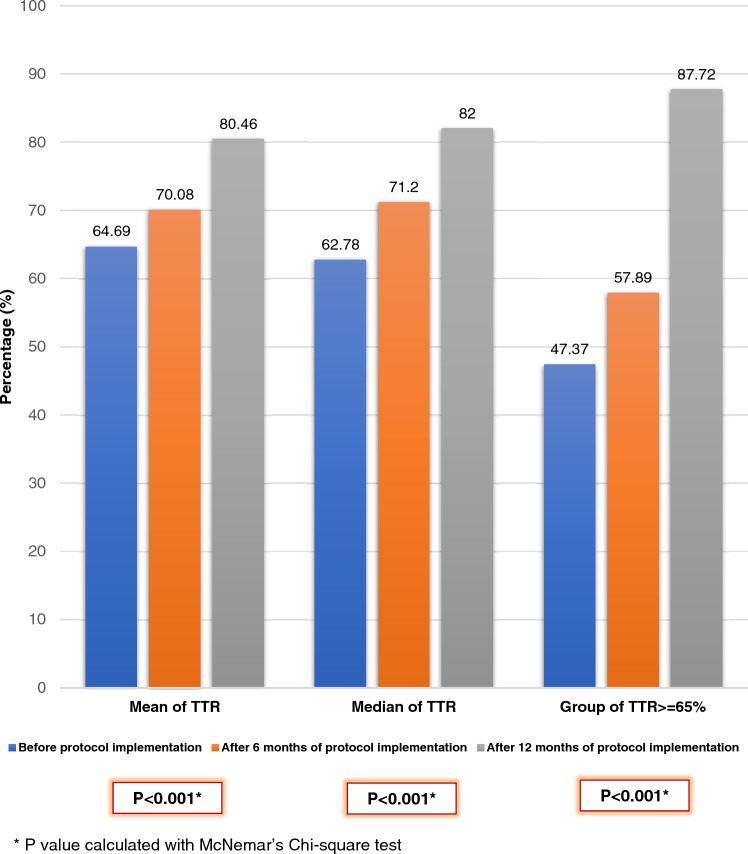
Change of TTR before and after 6 and 12 months of protocol implementation in 57 patients with non-valvular atrial fibrillation received warfarin.

Twelve months after the implementation of the protocol, there was a significant improvement in the TTR. The TTR improved to 80.46 ± 16.51 (*p* < 0.001), indicating a statistically significant increase. Moreover, there was a significant increase in the proportion of patients with a TTR of 65% or more. This proportion rose from 47 to 88% (*p* < 0.001). These improvements are detailed in Table 4 and illustrated in Figs. 1, 2, 3.

**Table 4:** Change of TTR after 6 and 12 months of protocol implementation compared between patients with TTR ≥ 65% and patients with TTR < 65%

TTR	Before protocol	After protocol 6 months	After protocol 12 months	*P*-value^1^
TTR (Mean ± SD) %	64.69 ± 20.14	70.08 ± 17.20	80.46 ± 16.51	** < 0.001**
TTR (Median) %	62.78	71.20	79.40	** < 0.001**
Group 1: TTR < 65%, n (%)	30 (52.63)	24 (42.11)	7 (12.28)	** < 0.001**
Group 2: TTR ≥ 65%, n (%)	27 (47.37)	33 (57.89)	50 (87.72)	** < 0.001**

^1^P-value for comparison between patients with TTR ≥ 65% and TTR < 65%

Significant values are in bold.

### Secondary outcomes

**Figure 4:**
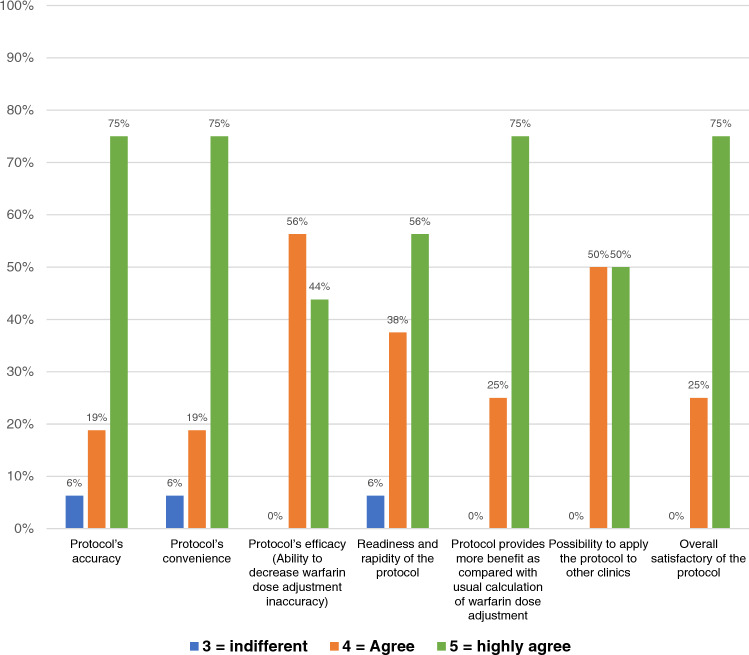
Bar chart showing satisfactory using TTR-INR guided warfarin adjustment protocol in patients with atrial fibrillation receiving vitamin K antagonist oral anticoagulant.

During the follow-up period, there were three recorded mortalities. Importantly, no ischemic or major bleeding events occurred in the first 12 months. For patients with a persistently low TTR of less than 55% despite adjustments to the protocol, a switch was made from VKAs to NOACs. This switch was made for ten patients. Only one patient was excluded due to a violation of the protocol. The feasibility of the protocol was assessed through a questionnaire. The responses indicated high levels of satisfaction and provided suggestions for improvement. These details are outlined in the manuscript and can be seen in Fig. 4 and Supplementary Appendix Table S6.

## Discussion

This study evaluated the effectiveness of a protocol for adjusting warfarin dosage. The protocol, guided by TTR and INR, was derived from the Hamilton Dosing Nomogram and Thai guidelines for patients with non-valvular atrial fibrillation (NVAF). After implementing the protocol for 12 months, the average TTR significantly increased from 65 to 80% (*p* < 0.001). Furthermore, the number of patients with a TTR of 65% or higher rose from 27 (47%) to 50 (88%) (*p* < 0.001), demonstrating the protocol’s effectiveness. Despite two rounds of warfarin adjustments according to the protocol, ten patients consistently had low TTR levels (below 65%). As a result, they were switched from VKA to NOACs due to their inability to reach the target INR and TTR levels (≥ 65%). This decision highlights the physicians’ meticulous approach to managing patients on warfarin therapy, as evidenced by regular assessments of INR and TTR levels at each visit. In addition, three patients were transitioned to NOACs due to difficulties in monitoring INR levels. Only one patient was excluded from the study due to a protocol violation, suggesting that the TTR-INR guided warfarin adjustment protocol is feasible and easy to use for most physicians. This is further supported by questionnaire responses, where the majority of participants either agreed or strongly agreed with items assessing the protocol’s feasibility.

Before the implementation of our protocol, only about 55% of patients achieved a TTR of 65% or higher. This aligns with a meta-analysis by Baker and colleagues, which highlighted the challenge of controlling INR levels among warfarin-receiving patients in the USA. Their study revealed that only 55% of AF patients in the USA maintained INR within the target range^10^. Similar trends were observed in Thailand. A study at Samutprakarn General Hospital^11^ involving 132 warfarin patients showed that only 22% achieved the target INR range of 2.0–3.0. Another study at Songklanagarind University Hospital found that only 32% of patients reached the target INR. Additional studies by Saokaew et al^12,13^. also showed varying TTR among AF patients in warfarin clinics, ranging from 31 to 56%.

Our study demonstrated a 15% increase in TTR after implementing the TTR-INR guided warfarin adjustment protocol for 12 months. However, clinical outcomes were not observed, possibly due to the short follow-up duration. Despite this, our findings support the recommendations from previous studies by Rosendaal et al.^14^, Witt et al.^15^, and Young et al.^16^, which advocated for systematic monitoring and adjustment of warfarin using protocols to improve TTR, clinical outcomes, and reduce complications.

During the follow-up, three deaths due to severe acute pancreatitis and sepsis were recorded. However, no treatment-related ischemic or significant bleeding events were observed in the initial 12 months, suggesting a favorable safety profile. This is consistent with a systematic review by Wan et al.^9^, which found that TTR could serve as a predictor of UAEs. A retrospective analysis of 21,540 patients revealed that a 7% increase in TTR was associated with a reduction in severe bleeding complications, while a 22% increase in TTR correlated with a decrease in ischemic events. Some clinical trials^9^ reported that every 10% increase in algorithm-consistent dosing of warfarin led to a 6.12% increase in TTR, accompanied by an 8% reduction in both ischemic stroke and severe bleeding complications.

### Study strength

Our study is a prospective study that utilizes a protocol-based approach for adjusting the warfarin dosage in patients with NVAF who are on warfarin as an anticoagulant. While TTR is a standard of care for patients with AF taking warfarin, its implementation is not widespread in Thailand. Notably, there was no prior study in Thailand that demonstrated the efficacy of using the Warfarin Dosing Nomogram, a TTR-INR guided warfarin adjustment protocol. As shown in numerous studies, algorithm-consistent dosing was effective, helped decrease human error in calculation, and was easy to use. The results of our study underscore the future use of protocol-based dose adjustment in Thailand, that potentially enhance the management of patients on warfarin therapy, improving their health outcomes and quality of life.

### Limitations

This study was a non-randomized, single-center investigation that collected data from patients at the Warfarin Clinic at King Chulalongkorn Memorial Hospital. The clinic staff, comprised of cardiologists, pharmacists, and nurses, were accustomed to warfarin and experienced in patient education about the drug. This expertise may have contributed to a higher baseline TTR compared to other studies from different centers or clinics.

The COVID-19 pandemic temporarily closed the clinic, which reopened in June 2021. This closure delayed patient recruitment and the calculation of the 12-month TTR. Further results are expected upon the study’s completion.

The pandemic also led to some patients being lost to follow-up or having to postpone scheduled visits with physicians. This may have introduced errors in the TTR calculation. Moreover, the study population did not meet the previously calculated sample size of 93 participants; instead, a total of 57 patients were analyzed for the primary outcome. Despite this, the data still showed a significantly large effect size in the improvement of TTR following the protocol implementation. This suggests the certainty of the results, even though the number of enrolled participants did not meet the previously calculated number.

### Future research

In order to gather more precise data and better reflect the real practice in Thailand, a prospective multicenter study of a similar design could be beneficial. This would allow for a larger and more diverse patient population from both the warfarin clinic and other clinics. Additionally, a longer follow-up period might provide more comprehensive data to elucidate clinical outcomes. This approach could potentially offer a more robust understanding of the effectiveness of the TTR-INR guided warfarin adjustment protocol in a real-world setting.

## Conclusion

This study provides valuable insights into the effectiveness and feasibility of a TTR-INR guided warfarin adjustment protocol in patients with NVAF. The implementation of this protocol resulted in substantial enhancements in TTR levels and the control of anticoagulation, without any observed complications related to the treatment during the first 12 months of follow-up. To confirm these findings and evaluate the impact of the protocol on clinical outcomes, further research involving larger patient groups and extended follow-up periods is recommended.

### Supplementary Information

Supplementary Figures.

Supplementary Tables.

## Data Availability

The authors confirm that the data supporting the findings of this study are available within the article and its supplementary materials.
