# Supplementary material for: Utility of TTR-INR guided warfarin adjustment protocol to improve time in therapeutic range in patients with atrial fibrillation receiving warfarin
Source: Sci Rep. 2024 May 22;14:11647. doi: 10.1038/s41598-024-61664-5 (PMC11109105; doi:10.1038/s41598-024-61664-5)
Supplement: Supplementary file 1 — Supplementary Figures. [file 41598_2024_61664_MOESM1_ESM.docx]

**Figure 1.** Individual change of TTR before and after 12 months of protocol implementation in
57 patients with non-valvular atrial fibrillation received warfarin

**Mean change of TTR = increase 16.81%**

**Figure 2.** Individual change of TTR before and after 6 and 12 months of protocol implementation in 57 patients with non-valvular atrial fibrillation received warfarin

**Figure 3.** Change of TTR before and after 6 and 12 months of protocol implementation in
57 patients with non-valvular atrial fibrillation received warfarin

**P<0.001***

**P<0.001***

**P<0.001***

* P value calculated with McNemar’s Chi-square test

**Figure 4.** Bar chart showing satisfactory using TTR-INR guided warfarin adjustment protocol in patients with atrial fibrillation receiving vitamin K antagonist oral anticoagulant.
