# Supplementary material for: Utility of TTR-INR guided warfarin adjustment protocol to improve time in therapeutic range in patients with atrial fibrillation receiving warfarin
Source: Sci Rep. 2024 May 22;14:11647. doi: 10.1038/s41598-024-61664-5 (PMC11109105; doi:10.1038/s41598-024-61664-5)
Supplement: Supplementary file 2 — Supplementary Tables. [file 41598_2024_61664_MOESM2_ESM.docx]

**Supplementary appendix**

The authors provide this appendix to give additional information for the readers.

**Table S1.** CHA2DS2-VASc score

**Table S2.** HAS-BLED score

**Table S3.** SAMe-TT2R2 score

**Table S4.** Definition of severity of bleeding

**Table S5.** TTR-INR guided warfarin adjustment protocol

**Table S6.** Bar chart showing satisfactory using TTR-INR guided warfarin adjustment protocol

**Table S1.** CHA2DS2-VASc score

|  | **Definition** | **Score** |
| --- | --- | --- |
| C | Congestive heart failure  Sign/symptoms of heart failure or objective evidence of reduce LVEF | 1 |
| H | Hypertension  Resting blood pressure > 140/90 mmHg on at least two occasions or current antihypertensive treatment | 1 |
| A2 | Age ≥ 75 years old | 2 |
| D | Diabetes mellitus  FPG > 125 mg/dL (7 mmol/L) or treatment with oral hypoglycemic agent and/or insulin | 1 |
| S2 | Previous stroke, transient ischemic attack, or thromboembolism | 2 |
| V | Vascular disease  Previous myocardial infarction, peripheral arterial disease, or aortic plaque | 1 |
| A | Age 65-74 years old | 1 |
| Sc | Sex category (Female) | 1 |
| **Maximum points** | | **9** |

**Table S2.** HAS-BLED score

|  | **Definition** | **Score** |
| --- | --- | --- |
| H | Hypertension (systolic blood pressure > 160 mmHg) | 1 |
| A | Abnormal renal and liver function^1^ | 1 or 2 |
| S | Stroke | 1 |
| B | Bleeding tendency/predisposition (anemia) | 1 |
| L | Labile INR (if on warfarin); time in therapeutic range < 60% | 1 |
| E | Elderly (age > 65 years old) | 1 |
| D | Drug or alcohol (antiplatelet or nonsteroidal anti-inflammatory drugs, or excess alcohol > 8 drinks/week) | 1 or 2 |
| **Maximum points** | | **9** |

^1^ **Abnormal renal function:** the presence of chronic dialysis, renal transplantation, serum creatinine > 200 mmol/L or serum creatinine > 2.26 mg/dL.

^1^ **Abnormal liver function:** chronic hepatic disease (e.g., cirrhosis) or biochemical evidence of significant hepatic derangement (bilirubin 2 to 3 times the upper limit of normal, in association with aspartate aminotransferase/alanine aminotransferase/alkaline phosphatase 3 times the upper limit normal).

**Table S3.** SAMe-TT2R2 score

|  | **Definition** | **Score** |
| --- | --- | --- |
| S | Sex (female) | 1 |
| A | Age < 60 years old | 1 |
| Me | Medical history^2^ | 1 |
| T | Treatment (interacting drugs, e.g., amiodarone for rhythm control) | 1 |
| T | Tobacco use (within 2 years) | 2 |
| R | Race (non-Caucasian) | 2 |
| **Maximum points** | | **8** |

^2^ Defined as more than 2 of following: hypertension, diabetes, coronary artery disease/myocardial infarction, peripheral arterial disease, congestive heart failure, previous stroke, pulmonary disease, and hepatic or renal disease.

**Table S4.** Definition severity of bleeding by International Society on Thrombosis
and Haemostasis (ISTH)

| **Definition of major bleeding (as fatal bleeding)** |
| --- |
| 1. Symptomatic bleeding in a critical area or organ such as intracranial, intraspinal, intraocular resulting in vision changes, retroperitoneal, intraarticular, pericardial, or intramuscular with compartment syndrome |
| 1. Bleeding causing a fall in Hb of ≥ 2 g/dL |
| 1. Bleeding leading to transfusion ≥ 2 units of whole blood or red cells |
| **Definition of Clinically relevant non-major bleeding (CRNM)** |
| - Acute or subacute clinically overt bleeding that did not satisfy the criteria for major bleeding and led to hospital admission for bleeding, physician-guided medical or surgical treatment for bleeding, or a change in antithrombotic therapy (including study drugs) for bleeding |
| **Definition of minor bleeding** |
| - All other bleeding (except major and CRNM) |

**Table S5.** TTR-INR guided warfarin adjustment protocol

**Follow-up Protocol**

1. Depend on TTR
2. Depend on INR and severity of bleeding

| **Previous TTR** | **INR** | **Bleeding** | **Follow-up** |
| --- | --- | --- | --- |
| ≥ 70 | < 1.5 | None | 1-2 weeks |
|  | 1.50-1.99 | None | 1-2 months |
|  | 2.00-3.00 | None | 3-4 months |
|  | 3.01-3.99 | None | 1-2 months |
|  | 4.00-4.99 | None/Minor bleeding | 1-2 weeks |
|  | 5.00-8.99 | None/Minor bleeding | 1 day |
|  | ≥ 9.00 | None/Minor bleeding | 1 day / admit |
|  | Any | Major bleeding | admit |
| 60-69 | < 1.5 | None | 1 week |
|  | 1.50-1.99 | None | 1 month |
|  | 2.00-3.00 | None | 2-3 months |
|  | 3.01-3.99 | None | 1 month |
|  | 4.00-4.99 | None/Minor bleeding | 1 week |
|  | 5.00-8.99 | None/Minor bleeding | 1 day |
|  | ≥ 9.00 | None/Minor bleeding | 1 day / admit |
|  | Any | Major bleeding | admit |
| < 60 | < 1.5 | None | 3 day – 1 week |
|  | 1.50-1.99 | None | 2 weeks – 1 month |
|  | 2.00-3.00 | None | 1-2 months |
|  | 3.01-3.99 | None | 2 weeks – 1 month |
|  | 4.00-4.99 | None/Minor bleeding | 3 days – 1 week |
|  | 5.00-8.99 | None/Minor bleeding | 1 day / admit |
|  | ≥ 9.00 | None/Minor bleeding | 1 day / admit |
|  | Any | Major bleeding | admit |

**Warfarin Dosing Nomogram (Warfarin dose 2 mg)**

| **Previous dose** | |  | **New dosing (depend on INR) (tab/week)** | | | | |  |
| --- | --- | --- | --- | --- | --- | --- | --- | --- |
| **mg/week** | **tab/week** |  | **≤ 1.5** | **1.51-1.99** | **2.00-3.00** | **3.01-3.99** | **4.00-4.99** |  |
|  |  |  |  |  |  |  |  |  |
| 6 | 3 |  | 3.5 | 3.5 | same | 2.5 | 2.5 |  |
| 7 | 3.5 |  | 4 | 4 | same | 3 | 2.5 |  |
| 8 | 4 |  | 4.5 | 4.5 | same | 3.5 | 3 |  |
| 9 | 4.5 |  | 5 | 5 | same | 4 | 3.5 |  |
| 10 | 5 |  | 6 | 5.5 | same | 4.5 | 4 |  |
| 11 | 5.5 |  | 6.5 | 6 | same | 5 | 4.5 |  |
| 12 | 6 |  | 7 | 6.5 | same | 5.5 | 5 |  |
| 13 | 6.5 |  | 7.5 | 7 | same | 6 | 5.5 |  |
| 14 | 7 |  | 8 | 7.5 | same | 6.5 | 6 |  |
| 15 | 7.5 |  | 9 | 8 | same | 7 | 6.5 |  |
| 16 | 8 |  | 9.5 | 8.5 | same | 7.5 | 7 |  |
| 17 | 8.5 |  | 10 | 9 | same | 8 | 7 |  |
| 18 | 9 |  | 10.5 | 9.5 | same | 8.5 | 7.5 |  |
| 19 | 9.5 |  | 11 | 10 | same | 9 | 8 |  |
| 20 | 10 |  | 12 | 11 | same | 9.5 | 8.5 |  |
| 21 | 10.5 |  | 12.5 | 11.5 | same | 9.5 | 9 |  |
| 22 | 11 |  | 13 | 12 | same | 10 | 9.5 |  |
| 23 | 11.5 |  | 13.5 | 12.5 | same | 10.5 | 10 |  |
| 24 | 12 |  | 14 | 13 | same | 11 | 10 |  |
| 25 | 12.5 |  | 15 | 13.5 | same | 11.5 | 10.5 |  |
| 26 | 13 |  | 15.5 | 14 | same | 12 | 11 |  |
| 27 | 13.5 |  | 16 | 14.5 | same | 12.5 | 11 |  |
| 28 | 14 |  | 16.5 | 15 | same | 13 | 12 |  |
| 29 | 14.5 |  | 17 | 15.5 | same | 13.5 | 12 |  |
| 30 | 15 |  | 18 | 16.5 | same | 14 | 13 |  |
| 31 | 15.5 |  | 18.5 | 17 | same | 14.5 | 13 |  |
| 32 | 16 |  | 19 | 17.5 | same | 15 | 13 |  |
| 33 | 16.5 |  | 19.5 | 18 | same | 15.5 | 13.5 |  |
| 34 | 17 |  | 20 | 18.5 | same | 16 | 14 |  |
| 35 | 17.5 |  | 21 | 19 | same | 16 | 14.5 |  |
| 36 | 18 |  | 21.5 | 19.5 | same | 16.5 | 15 |  |
| 37 | 18.5 |  | 22 | 20 | same | 17 | 15 |  |
| 38 | 19 |  | 22 | 20.5 | same | 17.5 | 15.5 |  |
| 39 | 19.5 |  | 23 | 21 | same | 18 | 16 |  |
| 40 | 20 |  | 24 | 22 | same | 18.5 | 16 |  |

**Warfarin Dosing Nomogram (Warfarin dose 3 mg)**

| **Previous dose** | |  | **New dosing (depend on INR) (tab/week)** | | | | |  |
| --- | --- | --- | --- | --- | --- | --- | --- | --- |
| **mg/week** | **tab/week** |  | **≤ 1.5** | **1.51-1.99** | **2.00-3.00** | **3.01-3.99** | **4.00-4.99** |  |
|  |  |  |  |  |  |  |  |  |
| 9 | 3 |  | 3.5 | 3.5 | same | 2.5 | 2.5 |  |
| 10.5 | 3.5 |  | 4 | 4 | same | 3 | 2.5 |  |
| 12 | 4 |  | 4.5 | 4.5 | same | 3.5 | 3 |  |
| 13.5 | 4.5 |  | 5 | 5 | same | 4 | 3.5 |  |
| 15 | 5 |  | 6 | 5.5 | same | 4.5 | 4 |  |
| 16.5 | 5.5 |  | 6.5 | 6 | same | 5 | 4.5 |  |
| 18 | 6 |  | 7 | 6.5 | same | 5.5 | 5 |  |
| 19.5 | 6.5 |  | 7.5 | 7 | same | 6 | 5.5 |  |
| 21 | 7 |  | 8 | 7.5 | same | 6.5 | 6 |  |
| 22.5 | 7.5 |  | 9 | 8 | same | 7 | 6.5 |  |
| 24 | 8 |  | 9.5 | 8.5 | same | 7.5 | 7 |  |
| 25.5 | 8.5 |  | 10 | 9 | same | 8 | 7 |  |
| 27 | 9 |  | 10.5 | 9.5 | same | 8.5 | 7.5 |  |
| 28.5 | 9.5 |  | 11 | 10 | same | 9 | 8 |  |
| 30 | 10 |  | 12 | 11 | same | 9.5 | 8.5 |  |
| 31.5 | 10.5 |  | 12.5 | 11.5 | same | 9.5 | 9 |  |
| 33 | 11 |  | 13 | 12 | same | 10 | 9.5 |  |
| 34.5 | 11.5 |  | 13.5 | 12.5 | same | 10.5 | 10 |  |
| 36 | 12 |  | 14 | 13 | same | 11 | 10 |  |
| 37.5 | 12.5 |  | 15 | 13.5 | same | 11.5 | 10.5 |  |
| 39 | 13 |  | 15.5 | 14 | same | 12 | 11 |  |
| 40.5 | 13.5 |  | 16 | 14.5 | same | 12.5 | 11 |  |
| 42 | 14 |  | 16.5 | 15 | same | 13 | 12 |  |
| 43.5 | 14.5 |  | 17 | 15.5 | same | 13.5 | 12 |  |
| 45 | 15 |  | 18 | 16.5 | same | 14 | 13 |  |
| 46.5 | 15.5 |  | 18.5 | 17 | same | 14.5 | 13 |  |
| 48 | 16 |  | 19 | 17.5 | same | 15 | 13 |  |
| 49.5 | 16.5 |  | 19.5 | 18 | same | 15.5 | 13.5 |  |
| 51 | 17 |  | 20 | 18.5 | same | 16 | 14 |  |
| 52.5 | 17.5 |  | 21 | 19 | same | 16 | 14.5 |  |
| 54 | 18 |  | 21.5 | 19.5 | same | 16.5 | 15 |  |
| 55.5 | 18.5 |  | 22 | 20 | same | 17 | 15 |  |
| 57 | 19 |  | 3.5 | 3.5 | same | 17.5 | 15.5 |  |
| 58.5 | 19.5 |  | 4 | 4 | same | 18 | 16 |  |
| 60 | 20 |  | 4.5 | 4.5 | same | 18.5 | 16 |  |

**Warfarin Dosing Nomogram (Warfarin dose 5 mg)**

| **Previous dose** | |  | **New dosing (depend on INR) (tab/week)** | | | | |  |
| --- | --- | --- | --- | --- | --- | --- | --- | --- |
| **mg/week** | **tab/week** |  | **≤ 1.5** | **1.51-1.99** | **2.00-3.00** | **3.01-3.99** | **4.00-4.99** |  |
|  |  |  |  |  |  |  |  |  |
| 15 | 3 |  | 3.5 | 3.5 | same | 2.5 | 2.5 |  |
| 17.5 | 3.5 |  | 4 | 4 | same | 3 | 2.5 |  |
| 20 | 4 |  | 4.5 | 4.5 | same | 3.5 | 3 |  |
| 22.5 | 4.5 |  | 5 | 5 | same | 4 | 3.5 |  |
| 25 | 5 |  | 6 | 5.5 | same | 4.5 | 4 |  |
| 27.5 | 5.5 |  | 6.5 | 6 | same | 5 | 4.5 |  |
| 30 | 6 |  | 7 | 6.5 | same | 5.5 | 5 |  |
| 32.5 | 6.5 |  | 7.5 | 7 | same | 6 | 5.5 |  |
| 35 | 7 |  | 8 | 7.5 | same | 6.5 | 6 |  |
| 37.5 | 7.5 |  | 9 | 8 | same | 7 | 6.5 |  |
| 40 | 8 |  | 9.5 | 8.5 | same | 7.5 | 7 |  |
| 42.5 | 8.5 |  | 10 | 9 | same | 8 | 7 |  |
| 45 | 9 |  | 10.5 | 9.5 | same | 8.5 | 7.5 |  |
| 47.5 | 9.5 |  | 11 | 10 | same | 9 | 8 |  |
| 50 | 10 |  | 12 | 11 | same | 9.5 | 8.5 |  |
| 52.5 | 10.5 |  | 12.5 | 11.5 | same | 9.5 | 9 |  |
| 55 | 11 |  | 13 | 12 | same | 10 | 9.5 |  |
| 57.5 | 11.5 |  | 13.5 | 12.5 | same | 10.5 | 10 |  |
| 60 | 12 |  | 14 | 13 | same | 11 | 10 |  |
| 62.5 | 12.5 |  | 15 | 13.5 | same | 11.5 | 10.5 |  |
| 65 | 13 |  | 15.5 | 14 | same | 12 | 11 |  |
| 67.5 | 13.5 |  | 16 | 14.5 | same | 12.5 | 11 |  |
| 70 | 14 |  | 16.5 | 15 | same | 13 | 12 |  |
| 72.5 | 14.5 |  | 17 | 15.5 | same | 13.5 | 12 |  |
| 75 | 15 |  | 18 | 16.5 | same | 14 | 13 |  |
| 77.5 | 15.5 |  | 18.5 | 17 | same | 14.5 | 13 |  |
| 80 | 16 |  | 19 | 17.5 | same | 15 | 13 |  |
| 82.5 | 16.5 |  | 19.5 | 18 | same | 15.5 | 13.5 |  |
| 85 | 17 |  | 20 | 18.5 | same | 16 | 14 |  |
| 87.5 | 17.5 |  | 21 | 19 | same | 16 | 14.5 |  |
| 90 | 18 |  | 21.5 | 19.5 | same | 16.5 | 15 |  |
| 92.5 | 18.5 |  | 22 | 20 | same | 17 | 15 |  |
| 95 | 19 |  | 22 | 20.5 | same | 17.5 | 15.5 |  |
| 97.5 | 19.5 |  | 23 | 21 | same | 18 | 16 |  |
| 100 | 20 |  | 24 | 22 | same | 18.5 | 16 |  |

**Warfarin Dosing Nomogram (Warfarin weekly pill schedule)**

| **Total tabs/week** | **Distribution in a week** | | | | | | |
| --- | --- | --- | --- | --- | --- | --- | --- |
|  | **Mon** | **Tue** | **Wed** | **Thu** | **Fri** | **Sat** | **Sun** |
| **3** | 0.5 | 0.5 | 0.5 | 0.5 | 0.5 | 0.5 | 0 |
| **3.5** | 0.5 | 0.5 | 0.5 | 0.5 | 0.5 | 0.5 | 0.5 |
| **4** | 0.5 | 0.5 | 0.5 | 0.5 | 0.5 | 0.5 | 1 |
| **4.5** | 0.5 | 1 | 0.5 | 0.5 | 1 | 0.5 | 0.5 |
| **5** | 1 | 0.5 | 1 | 0.5 | 1 | 0.5 | 0.5 |
| **5.5** | 0.5 | 1 | 0.5 | 1 | 0.5 | 1 | 1 |
| **6** | 1 | 0.5 | 1 | 1 | 0.5 | 1 | 1 |
| **6.5** | 1 | 1 | 1 | 1 | 1 | 1 | 0.5 |
| **7** | 1 | 1 | 1 | 1 | 1 | 1 | 1 |
| **7.5** | 1 | 1 | 1 | 1 | 1 | 1 | 1.5 |
| **8** | 1 | 1 | 1 | 1 | 1 | 1 | 2 |
| **8.5** | 1.5 | 1 | 1.5 | 1 | 1.5 | 1 | 1 |
| **9** | 1 | 2 | 1 | 1 | 2 | 1 | 1 |
| **9.5** | 1.5 | 1 | 1.5 | 1.5 | 1 | 1.5 | 1.5 |
| **10** | 1.5 | 1.5 | 1.5 | 1.5 | 1.5 | 1.5 | 1 |
| **10.5** | 1.5 | 1.5 | 1.5 | 1.5 | 1.5 | 1.5 | 1.5 |
| **11** | 1.5 | 1.5 | 1.5 | 1.5 | 1.5 | 1.5 | 2 |
| **11.5** | 1.5 | 2 | 1.5 | 1.5 | 2 | 1.5 | 1.5 |
| **12** | 2 | 1.5 | 2 | 1.5 | 2 | 1.5 | 1.5 |
| **12.5** | 1.5 | 2.5 | 1.5 | 1.5 | 2.5 | 1.5 | 1.5 |
| **13** | 2 | 1.5 | 2 | 2 | 1.5 | 2 | 2 |
| **13.5** | 2 | 2 | 2 | 2 | 2 | 2 | 1.5 |
| **14** | 2 | 2 | 2 | 2 | 2 | 2 | 2 |
| **14.5** | 2 | 2 | 2 | 2 | 2 | 2 | 2.5 |
| **15** | 2 | 2 | 2 | 2 | 2 | 2 | 3 |
| **15.5** | 2.5 | 2 | 2.5 | 2 | 2.5 | 2 | 2 |
| **16** | 2 | 3 | 2 | 2 | 3 | 2 | 2 |
| **16.5** | 2.5 | 2 | 2.5 | 2.5 | 2 | 2.5 | 2.5 |
| **17** | 2.5 | 2.5 | 2.5 | 2.5 | 2.5 | 2.5 | 2 |
| **17.5** | 2.5 | 2.5 | 2.5 | 2.5 | 2.5 | 2.5 | 2.5 |
| **18** | 2.5 | 2.5 | 2.5 | 2.5 | 2.5 | 2.5 | 3 |
| **18.5** | 2.5 | 3 | 2.5 | 2.5 | 3 | 2.5 | 2.5 |
| **19** | 3 | 2.5 | 3 | 2.5 | 3 | 2.5 | 2.5 |
| **19.5** | 2.5 | 3 | 2.5 | 3 | 2.5 | 3 | 3 |
| **20** | 3 | 2.5 | 3 | 3 | 2.5 | 3 | 3 |

**Table S6.** Bar chart showing satisfactory using TTR-INR guided warfarin adjustment protocol

Detail of satisfaction of Warfarin Dosing Nomogram was classified into numerical data, with a scale from 1 to 5 as followings: 1 = strongly disagree, 2 = disagree, 3 = indifferent, 4 = agree, and 5 = strongly agree, as showed in **figure 4**.

1. **Protocol’s accuracy:** 12 strongly agreed, 3 agreed, and 1 was indifferent.
2. **Protocol’s convenience:** 12 strongly agreed, 3 agreed, and 1 was indifferent.
3. **Protocol’s efficacy (Ability to decrease warfarin dose adjustment inaccuracy):** 9 strongly agreed, and 7 agreed.
4. **Readiness and rapidity of the protocol:** 9 strongly agreed, 6 agreed, and 1 was indifferent.
5. **Protocol provides more benefit as compared with usual calculation of warfarin dose adjustment:** 12 strongly agreed, and 4 agreed.
6. **Possibility to apply the protocol to other clinics:** 8 strongly agreed, and 8 agreed.
7. **Overall satisfactory of the protocol:** 12 were strongly satisfied, and 4 were satisfied.

There were also suggestions from the questionnaire responders, as follows:

1. The protocol makes it easier to adjust the drug dose.
2. This protocol is interesting method to adjust warfarin dose and should be printed as a hard copy at the physician’s table.
3. Developing the protocol into digital system, or application for smart phone should be considered.
4. The protocol makes it easier to follow and adjust warfarin to achieve target INR.
5. For some patients, adjustment of warfarin should be more personalized according to the patient’s conditions.
6. This study only included patients with NVAF, there might be possibilities to apply for patient taking warfarin for other indications.
7. There might be some protocol violation especially in circumstances which patient’s INR is remarkably close to target and prior INR was within target range. For example, a patient with INR of 1.97 and prior INR was in target range, warfarin dose should be increased according to protocol, but in real life the physician might not follow the protocol.
